# Gerosuppressant Metformin: less is more

**DOI:** 10.18632/aging.100316

**Published:** 2011-04-04

**Authors:** Javier A. Menendez, Sílvia Cufí, Cristina Oliveras-Ferraros, Luciano Vellon, Jorge Joven, Alejandro Vazquez-Martin

**Affiliations:** ^1^ Catalan Institute of Oncology, Girona (Catalonia, Spain); ^2^ Girona Biomedical Research Institute, Girona (Catalonia, Spain); ^3^ Fundación INBIOMED, Cell Reprogramming Unit, San Sebastián (Basque Country, Spain); ^4^ Centre de Recerca Biomèdica, Hospital Universitari Sant Joan de Reus, Institut d'Investigaciò Sanitària Pere Virgili, Universitat Rovira i Virgili, Reus (Catalonia, Spain)

## Less (mTOR signaling) is More (lifespan)

Further expanding the major findings of the landmark study by Harrison and colleagues showing that late-life pharmacological inhibition of the enzyme mammalian Target Of Rapamycin (mTOR) -a conserved integrator of nutrient and growth factor signaling- is sufficient to significantly extend lifespan in mice on rapamycin feeding [1], Selman and colleagues have recently revealed that loss of the ribosomal S6 protein kinase S6K1 -a downstream target and effector of mTOR- can similarly provide health and longevity effects without detrimental effects when targeted pharmacologically [2]. As previously observed in calorie restriction (CR)-related interventions that similarly delayed aging in Caenorhabditis elegans, rodents and rhesus monkeys [3], long-lived S6K1 knockout animals exhibit a reduced body size [2]. Pro-longevity features in animals lacking S6K1, including hyperactivation of the metabolic rheostat adenosine monophosphate (AMP)-activated protein kinase (AMPK) and transcriptional alterations of key genes involved in the regulation of glucose and lipid metabolism, paradoxically imitate those observed in reliable models of human progeria in which certain aspects of aging are manifested precociously or in exacerbated form [4]. We have learned that, in their attempt to re-allocate resources from growth to life extension, rapidly aging progeroid mice with nuclear envelope abnormalities -which could also be a central cause of normal aging- metabolically adopt CR-like strategies (i.e. activation of AMPK and inhibition of mTOR/S6K1 leading to extensive activation of autophagic catabolism) aimed to slow-down cellular division-dependent accumulation of genomic damage [5]. While it is obvious that systemic CR-like metabolic responses fail to repair nuclear architecture defects and rather accelerate death in progeroid mice, it is also obvious that, upon activation of AMPK in animals lacking S6K1, CR-like metabolic responses successfully accomplish an anti-aging mechanism while significantly decreasing body size [2].

## Less (AMPK-sensed anabolism) is More (lifespan)

If activation of AMPK (and/or inhibition of mTOR/S6K1) constitutes the best metabolic response to avoid that a normal growth rate would compromise somatic integrity in progeroid animals [4, 5], it is reasonable to suggest that the occurrence of AMPK upregulation upon loss of S6K1 would lead also to reductions in cell growth/division rates in order to avoid that bioenergetic stresses might trigger cell death. In this regard, we should acknowledge that metabolic requirements are identical in all proliferating (normal, cancer, and stem) cells and, proliferative metabolism is successfully adapted in all of them to facilitate and ensure the rapid uptake and efficient incorporation of nutrients into the biomass needed to produce new cell progeny [6]. An intuitive function of evolutionary conserved low-energy checkpoints such as the AMPK/mTOR/S6K1 longevity pathway is therefore to suppress cell proliferation by preventing nutrient utilization for anabolic processes [7, 8]. Unfortunately, it remains to be elucidated to which extent the interaction between the (AMPK-regulated) energy sensing and the (mTOR-regulated) sensing of signals arising from insulin, IGF-1, and amino-acids can integrate the effects of chronological aging with the effects of replicative aging in proliferative cells [9]. Moreover, the well-recognized ability of CR-induced deactivation of the mTOR pathway to slow growth, fitness and the onset of reproduction [10-12] while reducing risk of cancer (i.e. “preventing cancer by inhibiting aging” [13-15]) remains to be understood in terms of stem-cell contribution to homeostasis & repair in tissues with different degrees of cellular turnover and regenerative potential [9]. Although we know that anti-aging interventions started in younger animals appear to be more effective in terms of lifespan extension when compared to these interventions initiated in late middle or old age, a practical question that remains unanswered is: If started early in life, could CR-like interventions efficiently increase lifespan without interfering normal ontogenetic development?

Even if findings from Colman and colleagues demonstrating significant lifespan extension in rhesus monkeys can definitely substantiate CR as an effective pro-longevity strategy for humans [16], many people would prefer the effortlessness and perceived confidence of being treated with CR-Mimetic (CRM) pills. These CRMs are expected to reproduce some or all of the life- and health span-extending effects of CR including reduced incidence and delayed onset of age-related diseases such as cancer [17-20]. This new research on CRMs, therefore, aim to create or identify compounds that mimic CR effects by targeting metabolic (e.g. the glycolysis inhibitor 2-deoxyglucose) and stress response pathways (e.g. the sirtuin activator resveratrol) affected by CR, but without actually restricting caloric intake. Because promising results begin to emerge from initial studies regarding physiological responses which resemble those observed in CR (e.g. reduced plasma insulin), the insulin-sensitizing biguanide metformin begins to be catalogued as a CRM more than fifteen years after its launch for the treatment of type 2 diabetes. In vitro studies have suggested that metformin might molecularly recapitulate most of the pro-longevity effects occurring upon loss of S6K1. Metformin exposure efficiently suppresses S6K1 activity in cultured proliferating epithelial cells [21-23]. Despite overall reduction in global mRNA translation in response to metformin-induced dephosphorylation of S6K1 [24], metformin treatment triggers a complex transcriptional program that includes upregulation of genes coding for several aminoacyl-tRNA synthetases and RNA helicases [25], which might account for yet to be determined metformin-regulated mechanisms of differential translation with respect to specific mRNAs. A similar mechanism has been suggested by Selman and colleagues to explain the occurrence of AMPK protein upregulation upon loss of S6K1 [2]. If long-term exposure to the AMPK agonist metformin might activate a S6K1/AMPK auto-regulatory feed-back loop certainly merits to be investigated. Metformin treatment further affects the human transcriptome to modulate numerous genes coding for mitotic checkpoint proteins such as BubR1, which have also been reported to significantly impact on aging [26-28].

## Less (epithelial-to-mesenchymal transition) is More (somatic cell identity)

Metformin treatment has been recently found to selectively target tumor-initiating cancer stem cells (CSCs) in breast cancer cell cultures [29-31], likely mimicking a yet to be explored pro-longevity ability of metabolic stress-induced activation of AMPK [32]. On the one hand, a recently described transcriptional roadmap to the establishment of induced pluripotent stem (iPS) cells derived from somatic cells has revealed that induction of ground state pluripotency is favored by the inhibition of Epithelial-to-Mesenchymal Transition (EMT) and hence the induction of the reverse process, the Mesenchymal-to-Epithelial Transition (MET) [33]. On the other hand, during cell division, one of the major features of somatic cell reprogramming by defined factors, cells are potentially exposed to DNA damage. Inactivation of the Ataxia-telangiectasia mutated (ATM), which is critical in the cellular response to DNA double-strand breaks, has been recently shown to decrease reprogramming efficiency and leads to genomic instability in iPS cells [34]. Metformin treatment might directly impede the ontogeny of generating the CSC phenotype by repressing the stem cell property EMT [35-38]. Metformin treatment has been shown to transcriptional inhibit the expression of the pivotal inducer & drivers of the EMT machinery TGFβs, Zeb, Twist and Slug [35, 36]. Metformin treatment prevents conversion of epithelial into migratory mesenchymal cells by molecularly helping cancer cells to keep in their differentiated epithelial state [37]. Metformin's ability to enhance the epithelial behavior of cancer cells not only may occur through transcriptional inhibition of the “E-cadherin repressor interactome” (thus enhancing the expression of the metastasis suppressor protein E-cadherin at cell-cell junctions) but also by epigenetically preserving the differentiated phenotype of human epithelia via upregulation of the microRNA let-7a [38], a crucial regulator of CSC maintenance and point at connection between EMT and CSC formation [39]. Moreover, a previously unrecognized cancer chemopreventive activity of metformin (i.e. metformin's ability to cause pre-malignant lesions to recede or disappear thus preventing or delaying the onset of invasive cancer) may relate to metformin's ability to unexpectedly upregulate ATM activity in genomically unstable tumor cells [40-42]. Although there is no conclusive evidence that the maximal human lifespan is determined by declining stem-cell function or, conversely, that increasing the number of functionality of any single stem-cell population would extend lifespan, it would be relevant to evaluate whether metformin-activated CR-like molecular responses may raise reprogramming efficiency without increasing number of abnormal chromosomes during establishment of iPS cells. Given that EMT/MET and DNA-damage responses are typical events of development, tissue repair and tumor progression and considering the potential of iPS cells as a useful tool for elucidating the molecular mechanism of cell aging, forthcoming studies should clarify if metformin-induced repression of the mesenchymal program may actively regulate reprogramming of somatic cell identity, thus contributing to stem cell-related tissue homeostasis & repair (at least in tissues with high regenerative potential).

## Less (age at starting metformin treatment) is More (lifespan)

Translation of in vitro results into in vivo applications may significantly alter, however, the assumed CRM nature of the biguanide metformin. A recent study by Anisimov and colleagues published in a previous issue of Aging (Albany NY) may certainly establish that metformin should be defined as geroprotective or gerosuppressant rather than bona fide CRM. Long-living female mice from the outbred SHR strain were fed metformin in drinking water beginning at 3, 9 or 15 months of age and they were then analyzed for reproductive aging, mean & maximal lifespan and incidence of malignant tumors [43]. Metformin's effects on the reproductive function of SHR mice largely recapitulated metformin's ovulatory effects in women. Metformin is known to delay premature onset of the menstrual cycle in girls while restoring ovulations in women with premature menopause [44, 45]. Similarly, metformin treatment of SHR mice significantly improved reproductive function measured as a statistically significant increase in both the rate of SHR mice exhibiting shorter length of estrous cycles and the fraction of SHR mice with regular cycles. Prevention of reproductive aging (i.e. degeneration of the estrous cycle) occurred when metformin treatment was started at any age. These findings are consistent with a scenario in which a/the genetically-programmed function targeted by metformin (e.g. the mTOR pathway) is to drive reproduction because it was efficiently activated in an age-independent manner.

Earlier studies from the same group showed that metformin treatment increased mean lifespan of SHR mice by >37% and increased maximum lifespan by >10 months [46]. Metformin was found to extend the mean lifespan of the longest-living animals (i.e. the last 10% of survivors in the study population) by >20% and, in tumor-free mice, metformin treatment impressively extended the mean lifespan by ~70% (from 290 to 494 days). The incidences of the primary types of spontaneous malignant tumors in SHR mice (i.e. mammary carcinomas and leukemias), however, were not significantly affected by metformin feeding [46]. In their latest study, Anisimov and colleagues provide evidence that metformin fed late in life failed to extend lifespan in 15 month-old female SHR mice. Indeed, metfomin's ability to increase lifespan augments from an insignificant 6% when metformin treatment was initiated at the middle-age of 9 months up to 14% when metformin treatment was initiated early in life (i.e. at the young age of 3 months) [43]. In agreement with earlier findings in SHR mice [46], metformin treatment postponed detection of the first tumor by >20% when metformin feeding was initiated early and in the middle age; metformin treatment, however, failed to decrease total tumor incidence. In transgenic female mice (strain FVB/N) carrying a neu oncogene, which predispose animals to mammary cancer, the same group showed that metformin treatment modestly extended the lifespan by 8% and simultaneously delayed cancer onset and decreased cancer incidence & multiplicity [47, 48]. In female SHR mice, Anisimov and colleagues now confirm that metformin treatment, if started early in life, notably increases by 21% the mean lifespan of tumor-free mice [43]. In contrast, if started late in life, metformin treatment appears to significantly reduce (by 13%) the mean lifespan of tumor-free mice. These findings do not appear to support earlier suggestions that metformin might be better administered late in life to minimize early life metformin pleiotropic effects [e.g. metformin selected reservoir of malignancy-prone cells hypersensitive to selection of IGF-I signaling; 49-51]. It is perhaps relevant to note that, if started early in life, metformin treatment decreased the risk of death compared to the control group whereas similar treatment with metformin at older ages did not affect the relative risk of death in SHR female mice. Metformin's ability to increase the mean lifespan of tumor-free mice while simultaneously decreasing the risk of death in an age-related manner somewhat recapitulate metformin's ability to reduce cancer incidence among type 2 diabetic individuals. Confirming and expanding further a landmark study by Evans and colleagues [52], who originally reported that the risk of subsequent cancer diagnosis was significantly reduced in patients with type 2 diabetes who received metformin and that metformin's protective effect increased with greater metformin use (i.e. dose and/or time), a recently conducted retrospective study has reported an impressive 56% decrease in breast cancer risk among diabetic receiving metformin compared with diabetics treated with other therapies [53]. Available data reveal also that reductions in cancer mortality related to metformin use are similar in magnitude to reductions in cancer incidence, thus suggesting that metformin's anti-cancer effects largely depend on (or are restricted to) its preventive effects [54].

## Gerosuppressant metformin: Testing the “less is more” minimalist leitmotiv

If metformin both prevents cancer and extends lifespan in cancer-prone (e.g. neu-N transgenic mouse model of mammary cancer) strains of rodents, metformin's ability to prolong lifespan without affecting cancer in non-cancer-prone strain of rodents (e. g. SHR) may suggest that metformin can prolong life (and delay aging) by mechanisms unrelated to its ability to suppress cancer. It may not, however, if this discrepancy simply relies with the cellular response that oncogenes and tumor suppressors have on the phenotype of energetic metabolism. Derived from the FVB/N strain, neu-N transgenic mice express the nontransforming rat neu cDNA under the control of a mammary-specific promoter [55]. As a consequence, the mice develop spontaneous mammary adenocarcinomas beginning at approximately 125 days, with the majority of the mice haboring tumors by 300 days. This transgenic rodent model therefore resembles human breast cancer where neu is overexpressed, not mutated. Interestingly, the anabolic enzyme Fatty Acid Synthase (FASN) -an AMPK-related metformin's lipogenic target [56,57] - is differentially co-overexpressed with neu in the mammary epithelium of the neu-N female mice. Treatment of neu-N transgenic mice with the semi-synthetic inhibitor of FASN activity C75 has been found to significantly delay mammary tumor development (i.e. solely 20% of the C75-treated transgenic mice developed mammary carcinoma by 220 days compared to 50% in the vehicle control animals) and complete prevention of the disease in some neu-N animals [58]. This chemopreventive effect of the FASN inhibitor C75 related to the selective inhibition of both mammary duct and lobule development (i.e. C75 treatment significantly delayed mammary maturation as manifested by a reduction of both the number and the caliber of mammary ducts and budding epithelial structures) restricted to the neu-N transgenic mice. That is, pharmacological inhibition of FASN anabolic activity had no effect on mammary development in wild-type FVB/N mice. The fact that C75's ability to prevent mammary carcinomas via blockade of FASN-regulated lipogenesis appears to recapitulate metformin's cancer preventive effects in neu-N female mice suggests that metformin may have tumor suppressing and/or lifespan prolonging effects in response to specific metabolic phenotypes (e.g. high fuel intake, metabolic syndrome, diabetes and/or increased activation of oncogenic signals having similar effects on energy metabolism) but may have little or no effect at controlling cancer processes driven by energy-independent molecular events. Indeed, metformin has been found to attenuate and reverse the stimulatory effect of a high-energy diet on in vivo lung [59], breast [60] and colon carcinoma [61] growth, the latter occurring via metformin-induced inhibition of FASN expression. This complex scenario notably agrees with the suggestion that studies on cancer incidence and mortality among diabetics in relation to metformin use may not have implications for nondiabetics as they do not necessarily imply a universal metformin's chemopreventive action [54]. The available population studies are retrospective and confined to diabetics, in whom risk and prognostic factors that are relatively unimportant in the general population may have dominant roles [62]. Unfortunately, we are lacking even retrospective clinical evidence for antineoplastic activity in nondiabetics. Because the ideal cancer preventative strategy of the future would employ a limited course of low toxicity therapy to suppress pre-malignant lesions in high-risk cancer patients, forthcoming studies should provide a definitive support basis concerning clinical application of metformin and other biguanides as suppressors of broad-spectrum pre-malignant lesions. As recently suggested by Pollak [54], metformin-based large-scale cancer prevention trials would be more justifiable if we could provide criteria to specify high-risk populations in which metformin is expected to provide a greater benefit. Different experimental scenarios may provide crucial insights into metformin's ability to regulate the aging/cancer cross-paths:

## 1. Metformin and pre-malignant lesions

### Less (life-threatening invasive cancer) is More (cancer-free lifespan)

When considering recent studies describing that pre-malignant intraepithelial neoplasias such as ductal carcinoma in situ (DCIS) of the breast do contain pre-existing carcinoma precursor cells [63, 64], it would be of interest to evaluate whether metformin use in non-diabetic women might reduce the expected progression rate (12-15%) from non-invasive DCIS to life-threatening invasive breast cancer. In women over 40 years who did not have known breast cancer during life, the median prevalence of invasive breast cancer at death was 1.3% and the median prevalence of pre-invasive DCIS lesions was 8.9% in autopsy data [65]. These findings, together with the ability of progestins to reactivate CSCs in pre-existing breast carcinoma lesions, may explain why hormone replacement therapies significantly increase breast cancer risk in some women [66]. This metformin targetable reservoir of undetected, pre-invasive breast cancer lesions (or dormant CSCs within DCIS) could also underlie the apparent implausibility of increased breast cancer risk within 2 years among diabetic women receiving the insulin analogue glargine [67-69]. That is, hormonal factors influencing the natural history of some human malignancies including breast cancer (e.g. estrogen, insulin, IGF-1) could begin to manifest their effects in unexpected short timescales and the observed effects (i.e. rapid increase of breast cancer risk) are likely to reflect not the initiation of new tumors but rather the growth of subclinical malignant lesions to clinically diagnosable volumes [70]. Hence it would be reasonable to expect metformin to impose a strict control of the number and/or proliferation rate of tumor-initiating progenitors within intraepithelial neoplasias, which might be viewed as a very efficient preventive mechanism against invasive carcinomas including breast cancer in pre- and post-menopausal women. It should be noted that, when evaluating either how metformin efficacy on tumor growth and metastasis related to dietary energy availability or how metformin can inhibit the generation and/or maintenance of mammary tissue-specific stem cells and their niches, all the above mentioned studies observed or assumed that metformin treatment was highly effective at suppressing systemic metabolic biomarkers such as IGF-1, insulin, glucose or estradiol [59-61,71,72]. In Anisimov's experiments, however, metformin treatment was found to significantly decrease body temperature when its administration started at the age of 3 and 9 months but it failed to significantly affect levels of serum cholesterol, triglycerides, glucose and insulin. These findings, which evidently argue against the notion that metformin is a bona fide CRM, may intuitively suggest that metformin dose has been insufficient to alter cancer incidence despite being sufficient to alter other age-related pathologies (thus resulting in “partial” pro-healthy effects). It is noteworthy the recent data gained from a pilot clinical trial providing evidence that short-term, low-dose metformin (250 mg once daily for 1 month versus typical 500 mg three times daily in type 2 diabetes) safely and directly suppresses both colorectal epithelial proliferation and aberrant crypt formation [73]. Even acknowledged the fact that the gastrointestinal tract may be a special case in which metformin may act locally from the lumen following oral administration [74], we should wondering whether one can expect enhanced benefits by achieving more continuous exposure by using the slow-release metformin preparations developed for dosing convenience [75]. Would higher metformin doses achieve more impressive effects at increasing life span and/or postponing tumors in female SHR mice?

## 2. Metformin and genetic predisposition to breast cancer

### Less (endocrine-genotoxic liberation) is More (late survival of genetically-driven familial breast cancer)

It remains to be elucidated how metformin treatment, if started early and/or late in life, might significantly impact on late-onset diseases, like Alzheimer dementia, coronary artery diseases or, perhaps more importantly, certain familial forms of cancer. In this regard, an ideal scenario relates to genetic susceptibility to breast and ovarian cancer in women arising from a mutation in the BRCA1 gene, which is one of the most widespread genetic diseases [76, 77]. Women with a mutation have a 65% risk of developing breast cancer before age 70 [78]. Breast and ovarian cancers linked to mutations in BRCA1 are likely one of the main genetically-related causes of death in middle-age women and can be therefore regarded as important deleterious mutations in old age mortality [79]. Indeed, when all BRCA1 mutations are taken together, the prevalence of breast and ovarian cancer linked to the BRCA1 locus is one of the highest among late-onset diseases. Theoretical models of aging consider that selection pressures on survival at old ages is small (or even null after menopause for women) because survival at these ages no longer affects fitness. In this scenario, BRCA1 appears an excellent candidate gene for testing the impact of metformin on the theory of evolutionary theory of antagonist pleiotropy [80], for which the positive selection on alleles associated with increases of survival or fertility at young ages must be large enough to compensate for non-negligible negative selection on survival at old ages. Recent studies have suggested that variance in age of onset strongly influences selection of alleles involved in susceptibility to late-onset diseases that would lead to rapid death of carriers in the absence of modern medicine (e.g. breast cancer in BRCA1 carriers) and that, therefore, alleles under negative selection small enough to be traded-off for a positive effect at younger ages (e.g. BRCA1) may be more strongly negatively selected that thought [79]. Because CR itself is antagonistically pleiotropic (i.e. CR slows growth, development and reproduction -harmful early in life- and inhibits aging -beneficial later in life-), epidemiological and experimental approaches aimed to explore the impact of CR, CRMs and gerosuppressants such as metformin on the BRCA1-driven complex relationship between cancer & aging can be extremely informative. We should consider that BRCA1 is a large protein with multiple functional domains that interact directly or indirectly with a variety of molecules through which BRCA1 maintains genome integrity and represses tumor formation [81]. On the one hand, specific sequences of BRCA1 have been found to play a crucial role in longevity because their alteration results in the development of aging-like phenotypes including growth retardation and skin abnormalities [82]. On the other hand, BRCA1 deficiency leads to increase expression of several insulin-like growth factor (IGF) signalling axis members including IGF-I [83-86], a crucial target of the systemic effects of metformin [43, 71]. BRCA1 appears to directly recapitulate the AMPK-related anti-lipogenic effects of metformin (which rapidly induces inhibitory phosphorylation of Acetyl-CoA Carboxylase [ACACA] -the first committed molecule in the endogenous pathway of fatty acid biosynthesis-) because specific sequences of BRCA1 have been found to interact with and stabilize the phosphorylated form of ACACA, thus interfering with ACACA lipogenic activity by preventing ACACA dephosphorylation [87,88]. Because BRCA1 selectively inhibits aromatase expression and thus local, pro-tumorigenic estrogen production in breast adipose fibroblasts, breast adipose stromal cells and breast malignant epithelial cells [89-91], metformin's ability to concurrently inhibit IGF-1, endogenous lipogenesis and aromatase expression [92-94] must be carefully evaluated on its impact against the “endocrine-genotoxic liberation” [86] that occurs upon transfer from the wild-type to the mutant BRCA1 [86,95]. Further studies are warranted to evaluate if metformin treatment significantly modifies cancer risk due to deleterious BRCA1 gene mutations to decrease middle-age women mortality and/or late-onset familial cancer.

## 3. Metformin and the senescent phenotype

### 3.1. Less (accumulation of senescent cells) is More (activity of the immune system)

Anisimov and colleagues studied markers of cellular senescence in fibroblasts from skin of SHR mice treated with metformin since the 3rd and the 9th months of life [43]. Although no direct evidence is provided, the authors discuss the possibility that metformin treatment may actively prevent accumulation of senescent (“old”) cells. We should acknowledge that no hallmark of senescence thus far is entirely specific to the senescent state and that not all senescent cells express all possible senescence markers. Senescent cells, however, display several phenotypes that, altogether, can define the senescent state as recently reviewed by Rodier & Campisi [96]: 1.) Essentially irreversible growth arrest; 2.) significant increase in size; 3.) expression of senescence-associated β-galatosidase (SA-βgal; [97]) which reflects, at least in part, the increase in lysosomal mass [98]); 4.) expression of the tumor suppressor p16INK4a, which silences critical pro-proliferative genes by causing formation of senescence-associated heterochromatin foci (SAHF); 5.) persistent nuclear foci termed DNA segments with chromatin alterations reinforcing senescence (DNA-SCARS), which contain activated DNA damage response (DDR) proteins and include dysfunctional telomeres or telomere dysfunction-induced foci (TIF); and 6.) robust secretion of numerous growth factors, cytokines, proteases and other proteins that exhibit potent autocrine and paracrine activities (i.e. the senescence-associated secretory phenotype [SASP]). When some of theses hallmarks of senescent cells were qualitatively assessed in skin fibroblasts from SHR mice, metformin treatment was found to reduce the number of SAHF foci, the average nuclei area, and the nuclei accumulation of γH2AX compared to untreated control skin fibroblasts [99]. In addition, metformin treatment quantitatively reduced the number of fibroblasts with large nuclei area as well as the number of fibroblasts positive for SA-βgal activity and positive for intense nuclear γH2AX staining [99]. Because short-term dietary restriction has recently been shown to decrease abundance of senescent cells as assessed by assaying PCNA (as an indicator of DNA replication), γH2AX (as an indicator of DNA damage) and SA-βgal in the liver and intestine of middle-aged mice [100,101], and because γH2AX is a marker of mTOR-dependent cellular senescence in the absence of DNA damage and pharmacological blockade of mTOR activity with rapamycin efficiently decreases nuclear accumulation of γH2AX in senescent cells [102], it might be tempting to speculate that reduced cellular senescence might be a primary effect of metformin and, therefore, a causal, mTOR-regulated mechanism underlying metformin-increased lifespan in female SHR mice. If metformin treatment can function in a CR-like manner to actively prevent age-related accumulation of senescent cells and similar mechanism may apply to humans, it should be expected CR to increase the number of functional skin fibroblasts. We should acknowledge, however, that proliferation-competent skin fibroblasts did not increase after 9 to 12 years of CR in rhesus monkeys [103]. Nevertheless, it would be interesting to determine the impact of metformin treatment on the proportion of senescent cells in other tissues exquisitely sensitive to the structural and metabolic effects of CR such as the fat tissue [104-106]).

As mentioned above, one of the hallmarks of senescent cells is that the senescence growth arrest is essentially permanent and cannot be reversed by known physiological stimuli [96]. Therefore, metformin's ability to reduce the decrease senescent cell abundance as a function of total cell number may be understood mostly in terms of metformin-enhanced clearance of senescent cells by the immune system. Although it cannot be excluded that metformin treatment may actively inhibit production of new senescent cells (thus decreasing their “normal” frequency of accumulation in aging tissues) the fact that senescent cells are turned over slowly strongly suggests that metformin may enhance immune system responsiveness to senescent cells (thus increasing their “normal” clearance rate in aging tissues). If metformin treatment can restore tissue health by actively & selectively removing senescent cells in aging tissues via activation of the immune system, it can be expected that metformin-based interventions could delay age-related onset of chronic diseases more efficiently if started early in life because senescent cells' ability to induce macrophages declines with aging [104]. Anisimov's findings might support a metformin-triggered immunostimulatory-centered mechanism that may reduce age-related consequences of tissue inflammation due to senescent cell accumulation. But, do we have any evidence that metformin treatment promotes activation of the immune system? Long term CR has long been recognized to prevent the age-associated decrease in T-cell proliferative capacity in mice [107]. In nonhuman primates, CR has been found to similarly promote a significant improvement not only in the maintenance and/or production of naïve T cells but also in T cell function and reduced production of inflammatory cytokines by memory T cells [108]. More importantly, recent landmark studies have revealed that: a.) the metformin target mTOR is a major regulator of memory CD8 T-cell differentiation; b.) rapamycin has immunostimulatory effects on the generation of memory CD8 T cells, and c.) metformin itself enhances CD8 T-cell memory. Indeed, metformin-induced alteration in fatty acid metabolism during development of memory CD8 T cells has been found to considerable improve the efficacy of an anti-cancer vaccine [109-111]. Noteworthy, CD8 T cells, normally considered a part of adaptive immunity, also actively participate in innate immune responses.

### 3.2. Less (senescence bystander effect) is More (cancer progression-free interval)

Pioneeringly revealed by current Anisimov's studies, a previously unrecognized ability of metformin to regulate the senescent phenotype in vivo may provide crucial insights to definitely distinguish a/the molecular mechanisms underlying metformin-promoted anti-aging and/or anti-cancer effects. On the one hand, studies of human tissues and cancer-prone mice argue strongly that cellular senescence suppresses cancer in vivo [112-114]. Senescent cells can be found abundantly in intraepithelial pre-malignant lesions whereas senescent cells are scarce in invasive, life-threatening carcinomas. Dismantling the senescence response (e.g. by inactivating p53) causes a significant acceleration in the development of human tumors whereas the senescence response in established malignant states is associated with tumor regression. In this regard, the regressing tumor elicits an inflammatory response that stimulates the innate immune system, which eliminates the senescent cells. That is, an equivalent mechanism to the one proposed for metformin-induced clearance of senescent cells in the skin of female SHR mice. Age-related increases in senescent cells occur in mitotically competent tissues, which are those that give rise to cancer. Moreover, senescent cells have been shown to promote malignant progression of pre-malignant epithelial cells -which do not ordinarily form tumors- as well as established cancer cells in in vivo animal models [115-117]. Because many SASP proteins can alter epithelial cell differentiation, proliferation, migration & invasion (e.g. growth factors such as amphiregulin, cytokines such as IL-6/IL-8, VEGF, matrix metalloproteinases), a probable mechanism through which senescent cells can stimulate tumorigenesis in vivo is the bystander effect of senescent fibroblasts in the adjacent pre-malignant/malignant cells [96]. A critical test for the idea that senescent cells can promote the progression of age-related cancers might require strategies aimed to eliminate senescent cells or bystander effects of the SASP in vivo. Anisimov's findings (i.e. metformin treatment started early in life significantly postponed tumor formation in female SHR mice) appear to agree with the idea that metformin's ability to impair the genesis of senescent cells (by decreasing their generation, enhancing their elimination, or both) negatively impacts the progression of age-related cancers. Currently ongoing experiments in our laboratory aim to evaluate whether chronic exposure of non-transformed human fibroblasts to metformin significantly regulates replicative cellular senescence via prevention of proliferative exhaustion. Using cultured premalignant epithelial cells, we are evaluating also whether metformin treatment can prevent the hallmarks of malignancy EMT & invasiveness induced by SASP-containing conditioned media originated from senescent fibroblasts.

### 3.3. Less (threshold for stress-induced senescence) is More (elimination of oncogenically primed cells)

An unexplored scenario relates to metformin's ability to induce and/or enhance senescence responses in tumor cells themselves. Senescence-inducing stressors such as oxidative damage, DNA damage and/or oncogenes normally reach sufficient intensity to trigger senescence at the pre-malignant tumor stage. In agreement with a role of senescence in cancer prevention, the subsequent invasive progression of pre-malignant lesions almost inevitably involves one or more events that inhibit or impair the senescence pathway (e.g. convergence of EMT-driven acquisition of stem cell traits with enhanced autophagy in response to bioenergetic stress may turn pre-malignant phenotypes into cancer-initiating cells that efficiently couple invasive/metastatic spread to the bypass of metabolic stress- and oncogene-induced cellular senescence [63, 64]). Proliferative invasive cancer cells with activated oncogenes truly represent progeny of tumor cells that have acquired instrumental mechanisms to suppress senescence in earlier stages of cancer pathogenesis (e.g. in situ lesions). Therefore, activating the program of senescence in tumor cells seems an attractive approach to cancer treatment [118,119] and may help to molecularly explain the differential impact of metformin on aging and/or cancer in non-prone and cancer-prone animal models. We know that organisms in which cells fail to undergo senescence to not live longer; rather, they die prematurely of cancer [120]. Therefore, metformin treatment does not appear to impact aging and/or cancer by preventing general cellular senescence, which is rather necessary to communicate cellular damage/dysfunction to the surrounding tissue and stimulate repair, if needed. A normal senescence response helps to resolve fibrotic scars after acute liver injury whereas a compromised senescence response allows the occurrence of excessive, severe fibrosis [121-123]. Accordingly, excessive fibrosis due to a sustained, unresolved EMT-related synthesis of collagen and extracellular matrix synthesis can be found in many age-related human diseases such as chronic renal disease, non-alcoholic steatohepatitis, heart failure or sclerosis. Of note, we recently revealed the anti-fibrotic activity of metformin by using TGFβ-induced EMT in Madin-Darby canine kidney (MDCK) type I epithelial cells as an in vitro system to study the critical involvement of the EMT phenomenon in organ fibrosis [37]. Alternatively, metformin might be expected to block an essential senescence escape mechanism while nullifying EMT-driven CSC features by preventing molecular transition of epithelial tumour cells to embryonic mesenchymal phenotypes (i.e. EMT) [35-38]. Moreover, it may be relevant to evaluate whether metformin-altered cancer risk and improved health of laboratory animals may relate to its ability to activate DNA damage-like signaling to induce specific senescence-like growth inhibition of premalignant or malignant cells without altering normal breast structures or other non-neoplasic tissues. How?

Landmark studies of Lin *et al* [124] and Campaner *et al* [125] have recently found that the inhibition of the activity of cyclin dependent kinases (CDKs) plays a significant role in establishing protective cellular senescence; particularly, inhibition of CDK2 activity appears to be critical to lower the bar for triggering senescence in tumor cells [126]. Senescence-inducing stressors can inhibit CDK2 activity by either enhancing the expression of CDK2 inhibitory proteins (e.g. CDK inhibitory kinases or CKIs including p21Waf1/Cip1 and p27Kip1) or by inhibiting the expression of CDKi inhibitors such as Skp2, a component of the E3 ubiquitin ligase that mediates ubiquitin-dependent degradation of CKIs such as p27Kip1 (thus inhibiting CDK2 activity); in this CDK2/Skp2-inhibited scenario, oncogenically primed cells can be targeted by senescence early on or before reaching pre-tumoral or pre-malignant stages as occurs normally. Could metformin treatment “encourage cancer-prone cells to senesce” [126]? Although direct evidence for its occurrence is lacking, metformin may intriguingly function as a bona fide senescence-inducing stressor in cancer-prone cells when considering that: a.) metformin's inhibitory effects on tumor cell growth requires sufficient levels of CDKis (p21Waf1/Cip1 or p27Kip1) to bind and inhibit CDK2 [127]; b.) pharmacological and small inhibitory RNA-targeted ablation of FASN lipogenic activity -a well recognized metformin's molecular target- causes a dramatic down-regulation of Skp2 [128] to promote a prominent accumulation of p27Kip1 [128, 129]. Indeed, it might not be necessary for metformin to directly induce senescence in oncogenically primed cells. As reported by Lin *et al* [124], Skp2 inactivation on its own failed to induce cellular senescence; however, aberrant proto-oncogenic signals as well as inactivation of tumor suppressor genes triggered a potent, tumor-suppressive senescence response in mice and cells devoid of Skp2. It would be relevant to evaluate whether metformin co-exposure may favour the “accelerated senescence” triggered in normal cells by the expression of mutant Ras or Raf (oncogene-induced senescence) and by some other forms of supraphysiological mitogenic signaling [130-132] getting stuck irrespective of senescence-inhibiting adaptations (e.g. inactivation of p53). Because activation of a persistent DNA damage response (DDR), which initiates and maintains the senescence growth arrest, is a crucial anti-cancer barrier in early human tumorigenesis [133, 134], it would be very informative to test if metformin can significantly increase senescence cell abundance in premalignant lesions of the skin, the lung, and the pancreas [135]. Because epithelial cells within pre-malignant breast lesions that demonstrate markers of senescence and maintain an intact response to cellular stress identify women that are less likely to develop subsequent tumor events (i.e. the pro-senescent mechanisms of the Retinoblastoma (RB)/p16INK4a pathways are the most accurate predictors of recurrence and DCIS progression to EMT-enriched invasive basal-like breast carcinomas [136]), a molecular framework for “pro-senescence” metformin-based therapy would be evaluated in DCIS xenografts before and during in situ to invasive breast cancer transition [137-139]. Finally, because many tumor cells retain the ability to senesce, in culture and in vivo, in response to DNA-damaging chemotherapeutic agents [140-142], it might be tempting to suggest that metformin-enhanced cellular senescence in the context of DDR could explain metformin's ability to increase the rate of pathological complete responses to neoadjuvant chemotherapy in diabetic patients with breast cancer [143] and to promote tumor regression and prevent relapse when combined with sub-optimal doses of chemotherapy in animal models [144].

### 3.4. Less (cellular senescence-driven aging-like phenotypes) is More (retardation of premature aging)

Cellular senescence can drive are-related pathologies other than cancer. If metformin reduced abundance of senescent cells is central to metformin's ability to actively regulate lifespan extension, it would be interesting to test if metformin treatment may decelerate rapid accumulation of senescent cells and/or the rapid development of age-related phenotypes including growth retardation, loss of weight, lipodystrophy (i.e. loss of adiposity), hair loss and bone density defects. A mouse model of Hutchinson-Gilford progeria syndrome (HGPS; a childhood premature aging syndrome caused by the accumulation at the nuclear envelop of farnesylated forms of truncated prelamin A -a protein that is also altered during normal aging-) can be extremely informative because it develops phenotypes that overlap with those of HGPS children and do not include cancer even when causing a significant alteration in the number and proliferative capacity of epidermal stem cells [145, 146]. Administration of drugs interfering with the mevalonate pathway such as statins and aminobisphosphonates has been found to reduce DDR signaling in HGPS cells and to alleviate some of the progeroid symptoms in the HGPS mice [147]. Of note, in addition to ACACA, metformin's primary target AMPK has been shown to phosphorylate and thus regulate the activity of HMG-CoA reductase [148]. Metformin-induced activation of AMPK, like statins, can be expected to inhibit HMG-CoA reductase and suppress the production of downstream metabolites including dolichol and isoprenylated GTP-binding proteins. Indeed, metformin has been long recognized to inhibit cholesterol synthesis in diabetic rats and cultured fibroblasts via blockade of HMG-CoA reductase activity [149, 150]. It might be very instructive to evaluate whether metformin can replace statins and/or aminobisphosphonates in preventing aging-like phenotypes in animal models of HGPS, particularly because premature aging in mice involves a CR-like metabolic response molecularly equivalent to that occurring in situations reported to prolong lifespan. Thus, murine models of human HGPS exhibit an extensive basal activation of autophagy associated with a series of changes in lipid and glucose metabolic pathways leading to peripheral AMPK activation and mTOR inhibition [4, 5]. In the meantime, our laboratory is currently evaluating whether rapidly decreased growth-rate & premature entry into senescence of cultured HGPS fibroblasts is preventable by exogenous supplementation with metformin.

The key question that remains to be answered in Anisimov's studies relates to the molecular mechanism(s) through which metformin treatment increased lifespan and delayed tumor formation in female SHR mice. An ever-growing experimental body of evidence strongly suggests that metformin, at the cellular level, works as an efficient deactivator of the mTOR/S6K1gerogenetic pathway owing its ability to activate the metabolic rheostat AMPK [31]. Although we can speculate that metformin-imposed suppression of signaling through mTOR and S6K1 is the pivotal molecular event underlying metformin-induced promotion of longer lifespan & healthier aging, this possibility needs to be tested directly in future studies. What is obvious is that, if started early in life, chronic, sub-clinical doses of the putative mTOR/S6K1 deactivator metformin efficiently over-ride and take control over cell growth factors (gero-promoting) signaling to prevent aging-related tissue degeneration (e.g. metformin reduced abundance of senescent cells in the skin tissue) while improving reproductive function (e.g. metformin postponed age-related switch-off of the estrous cycle). Metformin's ability to prevent reproductive aging while increasing lifespan, if both occurring through AMPK activation-mTOR/S6K1 deactivation, appears to largely agree with a gerosuppressive functioning of metformin (i.e. gerosuppressants are pharmacological agents that inhibit intracellular signaling pathways that actively drive cellular aging and thus suppress the aging process [151,152]). Gerosuppressant metformin cannot distinguish between mTOR-driven program (which drives growth, fitness and the onset of reproduction early in life) and quasi-program (which causes menopauses, diseases such as cancer and aging later in life, when the mTOR-driven program becomes aimless) [151-153]. It is tempting to suggest that chronic exposure to sub-clinical doses of metformin institutes an autonomously-driven feedback regulatory loop of intermediary metabolism which ignores numerous & tissue-specific gero-promoting signaling to such a degree that it can significantly extend lifespan and postpone tumor formation. If we knew of any similar feedback inhibitor of mTOR that prevents age-related pathologies, analogies might then be helpful. Recent studies have revealed that sestrins -an ancient family of conserved oxidative proteins that accumulate in cells exposed to stress- activate AMPK and inhibit activation of mTOR. More importantly, sestrins appear to function as a negative feedback regulator of mTOR that integrates metabolic and stress inputs and prevents gero-pathologies caused by chronic activation of mTOR. Indeed, pharmacological activation of AMPK or inhibition of mTOR prevented age-associated pathologies induced by loss of sestrins [154-157]. In response to damaged mitochondrial production of reactive oxygen species (ROS), sestrin is upregulated to activate AMPK which in turn, inhibits mTOR to downregulate anabolism and promote mitochondrial biogenesis, thus decreasing ROS to feedback inhibiting sestrin. In response to metformin bound complex I of the respiratory chain electron transfer is inhibited and increases the levels of ROS [158]; inhibition of complex I would cause a decrease in energy supply that would in turn lead to a higher AMP/ATP ratio, and the concomitant activation of AMPK accompanied by downstream inhibition of mTOR; metformin-induced activation of AMPK increases the expression of peroxisome proliferator-activated response-gamma coactivator-1alpha (PGC-1alpha) and mitochondrial biogenesis [159, 160]. Metformin, as sestrins, can simultaneously function as ROS feedback homeostasis control system and as inhibitor of mTOR. As auspiciously suggested by Topisirovic & Sonemberg “as sestrins appear to amend the age-related effects of excessive TOR signaling, developing molecular mimics of sestrin could open new therapeutic avenues to target age-related pathologies” [155]. Results of forthcoming molecular biological studies should definitely establish if metformin is a “sestrin-mimic” molecule that clinically merits to be repositioned from type 2 diabetes to the tiny arsenal of anti-aging drugs.
